# Crystal structure of (*E*)-4-methyl-*N*-{2-[2-(4-nitro­benzyl­idene)hydrazin-1-yl]-2-oxoeth­yl}benzene­sulfonamide *N*,*N*-di­methyl­formamide monosolvate

**DOI:** 10.1107/S2056989017014669

**Published:** 2017-10-20

**Authors:** H. Purandara, Sabine Foro, B. Thimme Gowda

**Affiliations:** a Department of Chemistry, Mangalore University, Mangalagangotri 574 199, Mangalore, India; b Department of Chemistry, Sri Dharmasthala Manjunatheshwara College (Autonomous), Ujire, KA, India; c Institute of Materials Science, Darmstadt University of Technology, Alarich-Weiss-Strasse 2, D-64287, Darmstadt, Germany; d Karnataka State Rural Development and Panchayat Raj University, Gadag 582 101, Karnataka, India

**Keywords:** crystal structure, Schiff base, conformation, C—H⋯O hydrogen bond

## Abstract

The mol­ecule of the title Schiff base compound, C_16_H_16_N_4_O_5_S·C_3_H_7_NO, displays a *trans* conformation with respect to the C=N double bond. The C—N and N—N bonds are relatively short compared to their normal bond lengths, indicating some degree of delocalization in the mol­ecule. The mol­ecule is bent at the S atom, with an S—N—C—C torsion angle of 164.48 (11)°. The dihedral angle between the two aromatic rings is 84.594 (7)°. Inter­molecular N—H⋯O and C —H⋯O hydrogen bonds connect centrosymmetrically related mol­ecules into dimers forming rings of *R*_3_^3^(11) and *R*_2_^2^(10) graph-set motif stacked along the *a* axis into a columnar arrangement. The mol­ecular columns are further linked into a three-dimensional network by C—H⋯π inter­actions.

## Abstract 2

The title Schiff base mol­ecule displays a *trans* configurations with respect to the C=N double bond. Inter­molecular N—H⋯O and C —H⋯O hydrogen bonds connect centrosymmetrically related mol­ecules into dimers, forming rings of 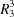
(11) and 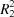
(10) graph-set motif stacked along the *a* axis into a columnar arrangement.

## Chemical context   

Hydrazones possess a wide variety of biological activities which include anti-inflammatory, analgesic, anti­convulsant, anti­tuberculous, anti­tumor, anti-HIV and anti­microbial activity. Hydrazones and their derivatives which can be prepared easily are stable and crystalline in nature. These characteristics have made them suitable compounds in recent times for drug design, ligands for metal complexes and for heterocyclic synthesis. Thus, hydrazones derived from *N*-(*p*-toluene­sulfon­yl)amino acids have been studied extensively for their biological and medicinal activities (Tian *et al.*, 2009 ▸, 2011 ▸; Shedid *et al.*, 2011 ▸). The inter­molecular inter­actions of *p*-toluene­sulfonyl­amide groups lead to supra­molecular structures. In continuation of our efforts to explore the potential of *N*-acyl­hydrazone derivatives, we report herein the synthesis and crystal structure of the title compound, (*E*)-4-methyl-*N*-{2-[2-(4-nitro­benzyl­idene)hydrazin-1-yl]-2-oxoeth­yl}benzene­sulfonamide *N*,*N*-di­methyl­formamide monosolvate.
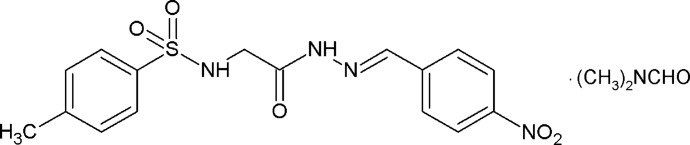


## Structural commentary   

**Figure 1:**
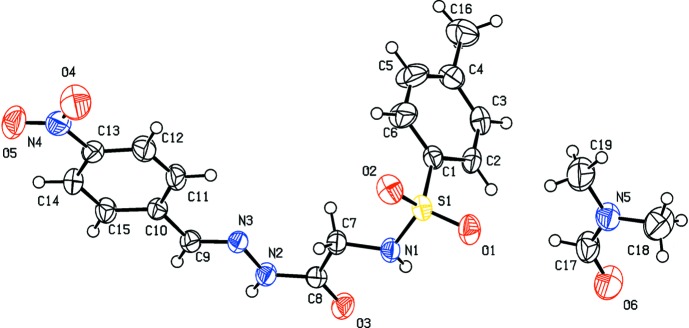
The mol­ecular structure of the title compound, with displacement ellipsoids drawn at the 50% probability level.

The title compound crystallizes as a di­methyl­formamide (DMF) monosolvate with one mol­ecule each of the Schiff base and solvent in the asymmetric unit (Fig. 1 ▸), and two mol­ecules in the unit cell (Fig. 3 ▸). The conformations of the C—H, N—H and C=O bonds in the central segment are *syn* to each other. The C8—O3 and C9—N3 bond lengths of 1.219 (2) and 1.274 (2) Å, respectively, confirm their significant double-bond characters. Further, the C8—N2 and N2—N3 bond lengths of 1.354 (2) and 1.3723 (18) Å, respectively, also indicate a significant delocalization of π-electron density over the hydrazone portion of the mol­ecule. The mol­ecule is bent at the S atom, with an S1—N1—C7—C8 torsion angle of 164.48 (11)°. The sulfonamide bond exists in a synclinal conformation, with a C—S—N—C torsion angle of −78.2 (1)°, which is the most preferred conformation for aromatic sulfon­amides (Katagiri *et al.*, 2014 ▸). The other central part of the mol­ecule is almost linear, as indicated by the C7—C8—N2—N3, C8—N2—N3—C9 and N2—N3—C9—C10 torsion angles [−1.6 (2), −178.98 (14) and 178.34 (13)°, respectively]. The relative orientation of the sulfonamide group with respect to the attached *p*-tolyl ring is given by the torsion angles C2—C1—S1—N1 = −79.45 (14)° and C6—C1—S1—N1 = 98.87 (16)°, while that of the hydrazone group with the attached 4-nitrobenzene ring is given by the torsion angles C11—C10—C9—N3 = 1.6 (2)° and C15—C10—C9—N3 = −177.27 (15)°, respectively. The dihedral angle between the C1–C6 sulfonyl benzene ring and the mean plane through the SO_2_—NH—CH_2_—CO segment is 81.452 (6)°, while that between the C10–C15 benzene ring and the plane through the C9—N3—N2—CO group is 4.296 (10)°. The dihedral angle between the two aromatic rings is 84.594 (7)°. The central part of the title compound, between atoms N1 and C9, is nearly planar with an extended chain conformation. The two benzene rings, *i.e.* C1–C6 and C10–C15, are inclined to the mean plane of the central spacer unit [O3/N1–N3/C7–C9; maximum deviation of 0.0353 (18) Å for C7] by 85.59 (8) and 4.35 (8)°, respectively.

## Supra­molecular features   

**Figure 2:**
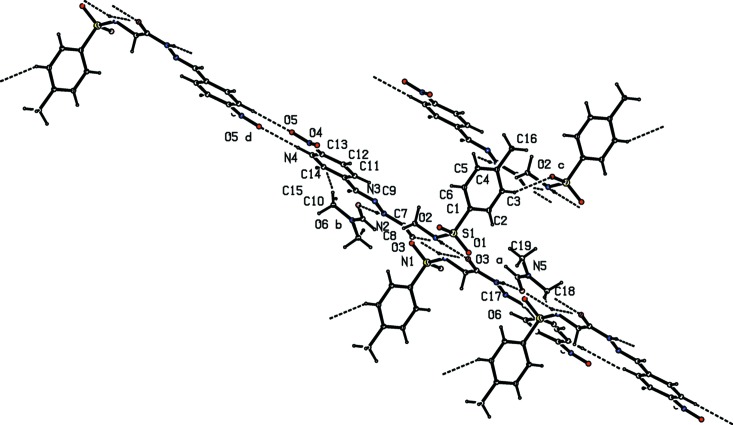
The hydrogen-bonding pattern (dashed lines) in the title compound.

**Figure 3:**
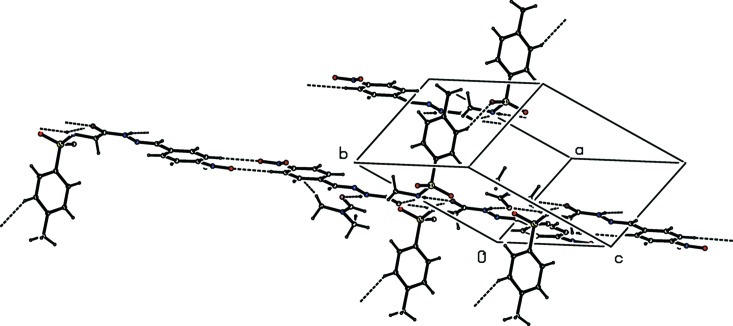
The mol­ecular packing of the title compound, with hydrogen bonding shown as dashed lines.

**Figure 4:**
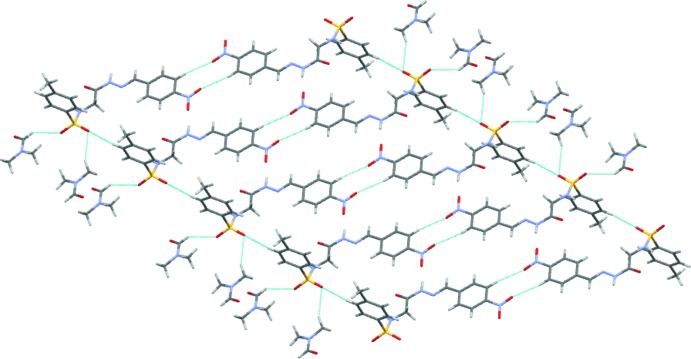
The C—H⋯O inter­actions (blue dotted lines) observed in the structure of the title compound

The Schiff base and solvent mol­ecules in the asymmetric unit are linked by N—H⋯O and C—H⋯O hydrogen bonds (Table 1 ▸ and Fig. 2 ▸), giving rise to a ring of 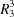
(11) graph-set motif. These bimolecular units are then linked by a pair of N—H⋯O hydrogen bonds, resulting in inversion dimers forming an 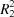
(10) ring motif (Fig. 3 ▸), which are linked into columns running parallel to the *a* axis by C—H⋯O hydrogen bonds involving aromatic C3 and sulfonyl O3 atoms (Fig. 4 ▸). Adjacent columns are further connected by C—H⋯π inter­actions, leading to the formation of a three-dimensional framework (Table 1 ▸).

## Database survey   

Comparison of the C—H⋯O inter­actions observed in the title compound, (I), with those of the 4-methyl derivative of *N*-acyl­hydrazone, namely (*E*)-*N*-{2-[2-(4-methyl­benzyl­idene)hydrazin-1-yl]-2-oxoeth­yl}-*p*-toluene­sulfonamide, (II) (Pur­andara *et al.*, 2015 ▸), indicates that the nitro group imparts a strong ability to the aromatic C—H groups to participate in C—H⋯O inter­actions, whereas the methyl substituent in the benzyl­idene ring of (II) does not activate aromatic protons for participating in inter­molecular C—H⋯O inter­actions. An aromatic H atom (C14—H14) of the nitro­phenyl moiety of (I) is involved in the formation of inter­molecular C—H⋯O inter­actions. The inductive effect of electron-withdrawing nitro group decreases the electronic density on the benzene ring. As a result, the nitro­phenyl moiety provides more acidic protons to form C—H⋯O hydrogen bonds.

## Synthesis and crystallization   

(*E*)-*N*-{2-[2-(4-Nitro­benzyl­idene)hydrazine-1-yl]-2-oxoeth­yl}-4-methyl­benzene­sulfonamide *N*,*N*-di­methyl­formamide mono­solvate was prepared as follows: *p*-toluene­sulfonyl chloride (0.01 mol) was added to glycine (0.02 mol) dissolved in an aqueous solution of potassium carbonate (0.06 mol, 50 ml). The reaction mixture was stirred at 373 K for 6 h, left overnight at room temperature, then filtered and treated with dilute hydro­chloric acid. The solid *N*-(4-methyl­benzene­sulfon­yl)glycine (*L*1) obtained was crystallized from aqueous ethanol. Sulfuric acid (0.5 ml) was added to *L*1 (0.02 mol) dissolved in ethanol (30 ml) and the mixture was refluxed. The reaction mixture was monitored by thin-layer chromatography (TLC) at regular inter­vals. After completion of the reaction, the reaction mixture was concentrated to remove excess ethanol. The product, *N*-(4-methyl­benzene­sulfon­yl)glycine ethyl ester (*L*2), was poured into water, neutralized with sodium bicarbonate and recrystallized from acetone. Pure *L*2 (0.01 mol) was then added in small portions to a stirred solution of 99% hydrazine hydrate (10 ml) in 30 ml ethanol and the mixture was refluxed for 6 h. After cooling to room temperature, the resulting precipitate was filtered, washed with cold water and dried to obtain *N*-(4-methyl­benzene­sulfon­yl)glycinyl hydrazide (*L*3). A mixture of *L*3 (0.01 mol) and *p*-nitro­benzaldehyde (0.01 mol) in anhydrous methanol (30 ml) and two drops of glacial acetic acid was refluxed for 8 h. After cooling, the precipitated (*E*)-*N*-{2-[2-(4-nitro­ben­zyl­idene)hydrazine-1-yl]-2-oxoeth­yl}-4-methyl­benzene­sul­fon­amide was collected by vacuum filtration, washed with cold methanol, dried and recrystallized to constant melting point from methanol (522–523 K). The purity of the compound was checked by TLC and characterized by its IR spectrum. The characteristic absorptions observed are 3236.6, 1687.7, 1587.4, 1338.6 and 1163.1 cm^−1^ for the stretching bands of N—H, C=O, C=N, S=O asymmetric and S=O symmetric, respectively. The characteristic ^1^H and ^13^C NMR specta of the title compound are as follows. ^1^H NMR (400 MHz, DMSO-*d*_6_: δ 2.36 (*s*, 3H), 3.61, 4.10 (*d*, 2H,), 7.36–7.39 (*m*, 2H, Ar—H), 7.72–7.74 (*m*, 2H, Ar—H), 7.86 (*d*, 2H, Ar—H), 8.23–8.27 (*m*, 2H, Ar—H), 7.93 (*t*, 1H), 8.02 (*s*, 1H), 11.74 (*s*, 1H). ^13^C NMR (400 MHz, DMSO-*d*_6_): δ 20.91, 43.20, 44.55, 123.94, 126.60, 127.81, 129.48, 137.48, 140.24, 141.40, 142.64, 144.62, 147.73, 164.64, 169.44. Prism-like colourless single crystals of the title compound employed in the X-ray diffraction study were grown from a DMF solution by slow evaporation of the solvent.

## Refinement   

Crystal data, data collection and structure refinement details are summarized in Table 2 ▸. H atoms bonded to C atoms were positioned with idealized geometry using a riding model, with C—H = 0.93 (aromatic), 0.96 (meth­yl) or 0.97 Å (methyl­ene). The amino H atoms were freely refined with the N—H distances restrained to 0.86 (2) Å. All H atoms were refined with isotropic displacement parameters set at 1.2*U*_eq_(C,N) or 1.5*U*_eq_(C) for methyl H atoms. A rotating model was used for the methyl groups.

## Supplementary Material

Crystal structure: contains datablock(s) I. DOI: 10.1107/S2056989017014669/rz5222sup1.cif

Structure factors: contains datablock(s) I. DOI: 10.1107/S2056989017014669/rz5222Isup2.hkl

CCDC reference: 1433602

Additional supporting information:  crystallographic information; 3D view; checkCIF report

## Figures and Tables

**Table 1:** Hydrogen-bond geometry (Å, °) *Cg*1 is the centroid of the C1–C6 ring.

*D*—H⋯*A*	*D*—H	H⋯*A*	*D*⋯*A*	*D*—H⋯*A*
N1—H1*N*⋯O3^i^	0.82 (2)	2.24 (2)	3.0142 (18)	158 (2)
N2—H2*N*⋯O6^i^	0.86 (2)	2.02 (2)	2.863 (2)	168 (2)
C3—H3⋯O2^ii^	0.93	2.59	3.442 (2)	152
C14—H14⋯O5^iii^	0.93	2.56	3.484 (2)	171
C18—H18*C*⋯O2^iv^	0.96	2.56	3.446 (3)	154
C15—H15⋯*Cg*1^v^	0.93	2.66	3.564 (2)	164

Symmetry codes: (i) 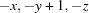; (ii) 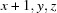; (iii) 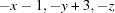; (iv) 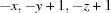; (v) 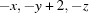.

**Table 2:** Experimental details

Crystal data	
Chemical formula	C_16_H_16_N_4_O_5_S·C_3_H_7_NO
*M*_r_	449.48
Crystal system, space group	Triclinic, *P* 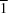
Temperature (K)	293
*a*, *b*, *c* (Å)	8.3515 (9), 10.5778 (9), 13.673 (1)
α, β, γ (°)	107.609 (7), 98.954 (8), 106.505 (8)
*V* (Å^3^)	1064.57 (18)
*Z*	2
Radiation type	Mo *K*α
μ (mm^−1^)	0.20
Crystal size (mm)	0.40 × 0.40 × 0.22

Data collection	
Diffractometer	Oxford Diffraction Xcalibur diffractometer with a Sapphire CCD detector
Absorption correction	Multi-scan (*CrysAlis RED*; Oxford Diffraction, 2009 ▸)
*T*_min_, *T*_max_	0.925, 0.958
No. of measured, independent and observed [*I* > 2σ(*I*)] reflections	7611, 4347, 3592
*R*_int_	0.010
(sin θ/λ)_max_ (Å^−1^)	0.625

Refinement	
*R*[*F* ^2^ > 2σ(*F* ^2^)], *wR*(*F* ^2^), *S*	0.039, 0.108, 1.04
No. of reflections	4347
No. of parameters	289
No. of restraints	2
H-atom treatment	H atoms treated by a mixture of independent and constrained refinement
Δρ_max_, Δρ_min_ (e Å^−3^)	0.32, −0.37

Computer programs: *CrysAlis CCD* and *CrysAlis RED* (Oxford Diffraction, 2009 ▸), *SHELXS97* and *SHELXTL* (Sheldrick, 2008 ▸), *SHELXL2014* (Sheldrick, 2015 ▸), *Mercury* (Macrae *et al.*, 2008 ▸) and *PLATON* (Spek, 2009 ▸).

## supplementary crystallographic information

### Crystal data

**Table:**

C_16_H_16_N_4_O_5_S·C_3_H_7_NO	*Z* = 2
*M**_r_* = 449.48	*F*(000) = 472
Triclinic, *P*1	*D*_x_ = 1.402 Mg m^−^^3^
*a* = 8.3515 (9) Å	Mo *K*α radiation, λ = 0.71073 Å
*b* = 10.5778 (9) Å	Cell parameters from 3760 reflections
*c* = 13.673 (1) Å	θ = 2.6–27.8°
α = 107.609 (7)°	µ = 0.20 mm^−^^1^
β = 98.954 (8)°	*T* = 293 K
γ = 106.505 (8)°	Prism, colourless
*V* = 1064.57 (18) Å^3^	0.40 × 0.40 × 0.22 mm

### Data collection

**Table:**

Oxford Diffraction Xcalibur single-crystal X-ray diffractometer with Sapphire CCD Detector	3592 reflections with *I* > 2σ(*I*)
Rotation method data acquisition using ω scans	*R*_int_ = 0.010
Absorption correction: multi-scan (CrysAlis RED; Oxford Diffraction, 2009)	θ_max_ = 26.4°, θ_min_ = 2.6°
*T*_min_ = 0.925, *T*_max_ = 0.958	*h* = −10→10
7611 measured reflections	*k* = −9→13
4347 independent reflections	*l* = −17→12

### Refinement

**Table:**

Refinement on *F*^2^	2 restraints
Least-squares matrix: full	Hydrogen site location: mixed
*R*[*F*^2^ > 2σ(*F*^2^)] = 0.039	H atoms treated by a mixture of independent and constrained refinement
*wR*(*F*^2^) = 0.108	*w* = 1/[σ^2^(*F*_o_^2^) + (0.0546*P*)^2^ + 0.3531*P*] where *P* = (*F*_o_^2^ + 2*F*_c_^2^)/3
*S* = 1.04	(Δ/σ)_max_ = 0.005
4347 reflections	Δρ_max_ = 0.32 e Å^−^^3^
289 parameters	Δρ_min_ = −0.37 e Å^−^^3^

### Special details

**Table:**

Geometry. All esds (except the esd in the dihedral angle between two l.s. planes) are estimated using the full covariance matrix. The cell esds are taken into account individually in the estimation of esds in distances, angles and torsion angles; correlations between esds in cell parameters are only used when they are defined by crystal symmetry. An approximate (isotropic) treatment of cell esds is used for estimating esds involving l.s. planes.

### Fractional atomic coordinates and isotropic or equivalent isotropic displacement parameters (Å^2^)

**Table:**

	*x*	*y*	*z*	*U*_iso_*/*U*_eq_	
C1	0.2529 (2)	0.80172 (17)	0.30177 (12)	0.0350 (3)	
C2	0.3764 (2)	0.73865 (18)	0.28367 (13)	0.0395 (4)	
H2	0.3431	0.6405	0.2546	0.047*	
C3	0.5479 (2)	0.8222 (2)	0.30897 (14)	0.0436 (4)	
H3	0.6298	0.7794	0.2971	0.052*	
C4	0.6013 (2)	0.9691 (2)	0.35194 (14)	0.0479 (4)	
C5	0.4745 (3)	1.0292 (2)	0.3654 (2)	0.0671 (6)	
H5	0.5067	1.1274	0.3914	0.080*	
C6	0.3022 (3)	0.9472 (2)	0.34106 (17)	0.0567 (5)	
H6	0.2198	0.9898	0.3511	0.068*	
C7	−0.0943 (2)	0.74344 (18)	0.10290 (13)	0.0385 (4)	
H7A	−0.0028	0.8356	0.1359	0.046*	
H7B	−0.1997	0.7535	0.1198	0.046*	
C8	−0.1211 (2)	0.69253 (16)	−0.01586 (13)	0.0348 (3)	
C9	−0.2478 (2)	0.96612 (17)	−0.05202 (13)	0.0356 (3)	
H9	−0.2613	0.9393	−0.1250	0.043*	
C10	−0.2839 (2)	1.09163 (16)	0.00553 (12)	0.0340 (3)	
C11	−0.2613 (3)	1.13753 (19)	0.11526 (14)	0.0476 (4)	
H11	−0.2174	1.0903	0.1539	0.057*	
C12	−0.3032 (3)	1.2520 (2)	0.16685 (14)	0.0503 (5)	
H12	−0.2892	1.2819	0.2400	0.060*	
C13	−0.3662 (2)	1.32173 (17)	0.10891 (13)	0.0386 (4)	
C14	−0.3851 (2)	1.28192 (18)	0.00117 (14)	0.0415 (4)	
H14	−0.4242	1.3322	−0.0364	0.050*	
C15	−0.3449 (2)	1.16557 (18)	−0.05016 (13)	0.0401 (4)	
H15	−0.3590	1.1364	−0.1233	0.048*	
C16	0.7899 (3)	1.0600 (3)	0.3827 (2)	0.0686 (6)	
H16A	0.8405	1.0282	0.3258	0.103*	
H16B	0.8491	1.0529	0.4458	0.103*	
H16C	0.8002	1.1568	0.3961	0.103*	
C17	0.2091 (3)	0.3550 (2)	0.33832 (16)	0.0609 (6)	
H17	0.1971	0.4207	0.3081	0.073*	
C18	0.2597 (4)	0.3016 (3)	0.49621 (18)	0.0765 (7)	
H18A	0.3792	0.3327	0.5340	0.115*	
H18B	0.2256	0.2072	0.4450	0.115*	
H18C	0.1896	0.3022	0.5456	0.115*	
C19	0.2526 (5)	0.5369 (3)	0.50861 (19)	0.0921 (10)	
H19A	0.2398	0.5910	0.4647	0.138*	
H19B	0.3644	0.5825	0.5590	0.138*	
H19C	0.1641	0.5308	0.5460	0.138*	
N1	−0.04825 (19)	0.64431 (15)	0.14518 (11)	0.0387 (3)	
H1N	−0.016 (2)	0.5851 (18)	0.1079 (14)	0.046*	
N2	−0.17143 (19)	0.77528 (14)	−0.06336 (11)	0.0385 (3)	
H2N	−0.181 (2)	0.758 (2)	−0.1298 (12)	0.046*	
N3	−0.19806 (17)	0.89339 (13)	−0.00295 (10)	0.0347 (3)	
N4	−0.4191 (2)	1.43967 (16)	0.16299 (13)	0.0497 (4)	
N5	0.2367 (2)	0.39543 (18)	0.44244 (12)	0.0535 (4)	
O1	0.02638 (17)	0.57237 (14)	0.29491 (11)	0.0516 (3)	
O2	−0.05327 (16)	0.78629 (15)	0.32153 (10)	0.0501 (3)	
O3	−0.09848 (18)	0.58545 (13)	−0.06656 (10)	0.0496 (3)	
O4	−0.4233 (3)	1.46197 (19)	0.25518 (13)	0.0808 (5)	
O5	−0.4588 (2)	1.50928 (16)	0.11362 (13)	0.0681 (4)	
O6	0.1980 (3)	0.23973 (17)	0.27815 (11)	0.0757 (5)	
S1	0.03312 (5)	0.69598 (5)	0.27126 (3)	0.03706 (13)	

### Atomic displacement parameters (Å^2^)

**Table:**

	*U*^11^	*U*^22^	*U*^33^	*U*^12^	*U*^13^	*U*^23^
C1	0.0402 (8)	0.0406 (9)	0.0286 (7)	0.0209 (7)	0.0080 (6)	0.0129 (6)
C2	0.0461 (9)	0.0387 (9)	0.0435 (9)	0.0246 (7)	0.0132 (7)	0.0187 (7)
C3	0.0430 (9)	0.0543 (11)	0.0461 (10)	0.0285 (8)	0.0133 (7)	0.0242 (8)
C4	0.0438 (10)	0.0525 (11)	0.0418 (9)	0.0167 (8)	0.0063 (7)	0.0125 (8)
C5	0.0575 (12)	0.0384 (10)	0.0833 (16)	0.0156 (9)	0.0118 (11)	−0.0032 (10)
C6	0.0489 (11)	0.0439 (10)	0.0701 (13)	0.0260 (9)	0.0140 (9)	0.0031 (9)
C7	0.0469 (9)	0.0381 (8)	0.0363 (8)	0.0223 (7)	0.0093 (7)	0.0155 (7)
C8	0.0335 (8)	0.0330 (8)	0.0363 (8)	0.0132 (6)	0.0063 (6)	0.0109 (7)
C9	0.0408 (8)	0.0348 (8)	0.0327 (8)	0.0136 (7)	0.0085 (6)	0.0146 (7)
C10	0.0348 (8)	0.0321 (8)	0.0361 (8)	0.0111 (6)	0.0076 (6)	0.0152 (7)
C11	0.0671 (12)	0.0460 (10)	0.0337 (9)	0.0289 (9)	0.0038 (8)	0.0161 (8)
C12	0.0694 (12)	0.0498 (11)	0.0303 (9)	0.0282 (9)	0.0057 (8)	0.0098 (8)
C13	0.0383 (8)	0.0332 (8)	0.0417 (9)	0.0137 (7)	0.0074 (7)	0.0104 (7)
C14	0.0469 (9)	0.0421 (9)	0.0454 (9)	0.0223 (8)	0.0120 (7)	0.0234 (8)
C15	0.0487 (9)	0.0440 (9)	0.0349 (8)	0.0205 (8)	0.0122 (7)	0.0197 (7)
C16	0.0492 (12)	0.0686 (14)	0.0714 (15)	0.0119 (10)	0.0067 (10)	0.0157 (12)
C17	0.0906 (16)	0.0637 (13)	0.0425 (10)	0.0440 (12)	0.0159 (10)	0.0236 (10)
C18	0.125 (2)	0.0649 (14)	0.0463 (12)	0.0357 (15)	0.0264 (13)	0.0263 (11)
C19	0.148 (3)	0.0744 (16)	0.0518 (14)	0.0653 (18)	0.0017 (15)	0.0082 (12)
N1	0.0462 (8)	0.0389 (8)	0.0369 (8)	0.0245 (6)	0.0084 (6)	0.0143 (6)
N2	0.0517 (8)	0.0368 (7)	0.0301 (7)	0.0211 (6)	0.0092 (6)	0.0120 (6)
N3	0.0391 (7)	0.0319 (7)	0.0340 (7)	0.0150 (6)	0.0077 (5)	0.0118 (6)
N4	0.0467 (9)	0.0446 (9)	0.0519 (10)	0.0204 (7)	0.0073 (7)	0.0085 (7)
N5	0.0735 (11)	0.0551 (9)	0.0376 (8)	0.0337 (9)	0.0124 (7)	0.0157 (7)
O1	0.0566 (8)	0.0575 (8)	0.0568 (8)	0.0243 (6)	0.0169 (6)	0.0377 (7)
O2	0.0494 (7)	0.0667 (8)	0.0454 (7)	0.0334 (6)	0.0216 (6)	0.0190 (6)
O3	0.0665 (8)	0.0425 (7)	0.0430 (7)	0.0318 (6)	0.0107 (6)	0.0099 (5)
O4	0.1144 (14)	0.0879 (12)	0.0502 (9)	0.0643 (11)	0.0239 (9)	0.0111 (8)
O5	0.0834 (11)	0.0599 (9)	0.0783 (10)	0.0475 (8)	0.0219 (8)	0.0277 (8)
O6	0.1306 (15)	0.0703 (10)	0.0394 (8)	0.0556 (10)	0.0250 (8)	0.0178 (7)
S1	0.0401 (2)	0.0458 (2)	0.0349 (2)	0.02253 (18)	0.01295 (16)	0.01948 (18)

### Geometric parameters (Å, º)

**Table:**

C1—C6	1.377 (2)	C13—C14	1.374 (2)
C1—C2	1.393 (2)	C13—N4	1.468 (2)
C1—S1	1.7629 (17)	C14—C15	1.381 (2)
C2—C3	1.376 (2)	C14—H14	0.9300
C2—H2	0.9300	C15—H15	0.9300
C3—C4	1.389 (3)	C16—H16A	0.9600
C3—H3	0.9300	C16—H16B	0.9600
C4—C5	1.390 (3)	C16—H16C	0.9600
C4—C16	1.507 (3)	C17—O6	1.215 (2)
C5—C6	1.379 (3)	C17—N5	1.318 (2)
C5—H5	0.9300	C17—H17	0.9300
C6—H6	0.9300	C18—N5	1.438 (3)
C7—N1	1.452 (2)	C18—H18A	0.9600
C7—C8	1.505 (2)	C18—H18B	0.9600
C7—H7A	0.9700	C18—H18C	0.9600
C7—H7B	0.9700	C19—N5	1.451 (3)
C8—O3	1.2191 (19)	C19—H19A	0.9600
C8—N2	1.354 (2)	C19—H19B	0.9600
C9—N3	1.274 (2)	C19—H19C	0.9600
C9—C10	1.464 (2)	N1—S1	1.6082 (14)
C9—H9	0.9300	N1—H1N	0.818 (15)
C10—C15	1.384 (2)	N2—N3	1.3723 (18)
C10—C11	1.393 (2)	N2—H2N	0.856 (15)
C11—C12	1.374 (3)	N4—O5	1.216 (2)
C11—H11	0.9300	N4—O4	1.218 (2)
C12—C13	1.376 (2)	O1—S1	1.4273 (13)
C12—H12	0.9300	O2—S1	1.4333 (13)
			
C6—C1—C2	119.83 (16)	C15—C14—H14	120.7
C6—C1—S1	119.93 (13)	C14—C15—C10	121.03 (16)
C2—C1—S1	120.22 (13)	C14—C15—H15	119.5
C3—C2—C1	119.72 (16)	C10—C15—H15	119.5
C3—C2—H2	120.1	C4—C16—H16A	109.5
C1—C2—H2	120.1	C4—C16—H16B	109.5
C2—C3—C4	121.40 (16)	H16A—C16—H16B	109.5
C2—C3—H3	119.3	C4—C16—H16C	109.5
C4—C3—H3	119.3	H16A—C16—H16C	109.5
C3—C4—C5	117.63 (18)	H16B—C16—H16C	109.5
C3—C4—C16	121.21 (18)	O6—C17—N5	126.0 (2)
C5—C4—C16	121.16 (19)	O6—C17—H17	117.0
C6—C5—C4	121.71 (18)	N5—C17—H17	117.0
C6—C5—H5	119.1	N5—C18—H18A	109.5
C4—C5—H5	119.1	N5—C18—H18B	109.5
C1—C6—C5	119.63 (17)	H18A—C18—H18B	109.5
C1—C6—H6	120.2	N5—C18—H18C	109.5
C5—C6—H6	120.2	H18A—C18—H18C	109.5
N1—C7—C8	110.67 (13)	H18B—C18—H18C	109.5
N1—C7—H7A	109.5	N5—C19—H19A	109.5
C8—C7—H7A	109.5	N5—C19—H19B	109.5
N1—C7—H7B	109.5	H19A—C19—H19B	109.5
C8—C7—H7B	109.5	N5—C19—H19C	109.5
H7A—C7—H7B	108.1	H19A—C19—H19C	109.5
O3—C8—N2	121.73 (15)	H19B—C19—H19C	109.5
O3—C8—C7	123.18 (14)	C7—N1—S1	118.51 (11)
N2—C8—C7	115.08 (13)	C7—N1—H1N	118.6 (14)
N3—C9—C10	120.54 (14)	S1—N1—H1N	115.5 (14)
N3—C9—H9	119.7	C8—N2—N3	119.45 (13)
C10—C9—H9	119.7	C8—N2—H2N	120.9 (13)
C15—C10—C11	118.91 (15)	N3—N2—H2N	119.5 (13)
C15—C10—C9	119.51 (14)	C9—N3—N2	116.76 (13)
C11—C10—C9	121.57 (14)	O5—N4—O4	123.01 (17)
C12—C11—C10	120.49 (16)	O5—N4—C13	118.74 (16)
C12—C11—H11	119.8	O4—N4—C13	118.25 (16)
C10—C11—H11	119.8	C17—N5—C18	120.84 (18)
C11—C12—C13	119.13 (16)	C17—N5—C19	122.27 (18)
C11—C12—H12	120.4	C18—N5—C19	116.84 (17)
C13—C12—H12	120.4	O1—S1—O2	119.85 (8)
C14—C13—C12	121.87 (16)	O1—S1—N1	107.10 (8)
C14—C13—N4	118.89 (15)	O2—S1—N1	106.47 (8)
C12—C13—N4	119.22 (16)	O1—S1—C1	107.68 (8)
C13—C14—C15	118.52 (15)	O2—S1—C1	107.05 (8)
C13—C14—H14	120.7	N1—S1—C1	108.25 (8)
			
C6—C1—C2—C3	2.5 (3)	C11—C10—C15—C14	−0.8 (3)
S1—C1—C2—C3	−179.22 (12)	C9—C10—C15—C14	178.07 (15)
C1—C2—C3—C4	−0.4 (3)	C8—C7—N1—S1	164.48 (11)
C2—C3—C4—C5	−2.1 (3)	O3—C8—N2—N3	178.85 (15)
C2—C3—C4—C16	178.09 (18)	C7—C8—N2—N3	−1.6 (2)
C3—C4—C5—C6	2.5 (3)	C10—C9—N3—N2	178.34 (13)
C16—C4—C5—C6	−177.7 (2)	C8—N2—N3—C9	−178.98 (14)
C2—C1—C6—C5	−2.1 (3)	C14—C13—N4—O5	−8.6 (3)
S1—C1—C6—C5	179.61 (17)	C12—C13—N4—O5	173.06 (18)
C4—C5—C6—C1	−0.4 (4)	C14—C13—N4—O4	170.45 (18)
N1—C7—C8—O3	−3.0 (2)	C12—C13—N4—O4	−7.9 (3)
N1—C7—C8—N2	177.44 (14)	O6—C17—N5—C18	1.0 (4)
N3—C9—C10—C15	−177.27 (15)	O6—C17—N5—C19	178.4 (3)
N3—C9—C10—C11	1.6 (2)	C7—N1—S1—O1	165.96 (13)
C15—C10—C11—C12	1.7 (3)	C7—N1—S1—O2	36.61 (15)
C9—C10—C11—C12	−177.14 (17)	C7—N1—S1—C1	−78.19 (14)
C10—C11—C12—C13	−0.7 (3)	C6—C1—S1—O1	−145.65 (15)
C11—C12—C13—C14	−1.3 (3)	C2—C1—S1—O1	36.03 (15)
C11—C12—C13—N4	177.02 (17)	C6—C1—S1—O2	−15.55 (17)
C12—C13—C14—C15	2.2 (3)	C2—C1—S1—O2	166.13 (13)
N4—C13—C14—C15	−176.14 (15)	C6—C1—S1—N1	98.87 (16)
C13—C14—C15—C10	−1.1 (3)	C2—C1—S1—N1	−79.45 (14)

### Hydrogen-bond geometry (Å, º)

Cg1 is the centroid of the C1–C6 ring.

**Table:**

*D*—H···*A*	*D*—H	H···*A*	*D*···*A*	*D*—H···*A*
N1—H1*N*···O3^i^	0.82 (2)	2.24 (2)	3.0142 (18)	158 (2)
N2—H2*N*···O6^i^	0.86 (2)	2.02 (2)	2.863 (2)	168 (2)
C3—H3···O2^ii^	0.93	2.59	3.442 (2)	152
C14—H14···O5^iii^	0.93	2.56	3.484 (2)	171
C18—H18*C*···O2^iv^	0.96	2.56	3.446 (3)	154
C15—H15···*Cg*1^v^	0.93	2.66	3.564 (2)	164

Symmetry codes: (i) −*x*, −*y*+1, −*z*; (ii) *x*+1, *y*, *z*; (iii) −*x*−1, −*y*+3, −*z*; (iv) −*x*, −*y*+1, −*z*+1; (v) −*x*, −*y*+2, −*z*.
